# X-chromosome terminal deletion in a female with premature ovarian failure: Haploinsufficiency of X-linked genes as a possible explanation

**DOI:** 10.1186/1755-8166-3-14

**Published:** 2010-07-20

**Authors:** Susana I Ferreira, Eunice Matoso, Marta Pinto, Joana Almeida, Thomas Liehr, Joana B Melo, Isabel M Carreira

**Affiliations:** 1 Laboratório de Citogenética, Instituto de Biologia Médica, Faculdade de Medicina, Universidade de Coimbra, 3000-354 Coimbra, Portugal; 2 Serviço de Ginecologia, Maternidade Bissaya Barreto, 3000-061 Coimbra, Portugal; 3 Jena University Hospital, Institute of Human Genetics and Anthropology, D-007740 Jena, Germany; 4 CIMAGO, Universidade de Coimbra, 3001-301 Coimbra, Portugal; 5 Centro de Neurociências e Biologia Celular, Universidade de Coimbra, 3000-354 Coimbra, Portugal

## Abstract

**Background:**

Premature ovarian failure (POF) has repeatedly been associated to X-chromosome deletions. *FMR1 *gene premutation allele's carrier women have an increased risk for POF. We intent to determine the cause of POF in a 29 year old female, evaluating both of these situations.

**Methods:**

Concomitant analysis of *FMR1 *gene CGG repeat number and karyotype revealed an X-chromosome terminal deletion. Fluorescence *in situ *further characterized the breakpoint. A methylation assay for *FMR1 *gene allowed to determine its methylation status, and hence, the methylation status of the normal X-chromosome.

**Results:**

We report a POF patient with a 46,X,del(X)(q26) karyotype and with skewed X-chromosome inactivation of the structural abnormal X-chromosome.

**Conclusions:**

Despite the hemizygosity of *FMR1 *gene, the patient does not present Fragile X syndrome features, since the normal X-chromosome is not subject to methylation. The described deletion supports the hypothesis that haploinsufficiency of X-linked genes can be on the basis of POF, and special attention should be paid to X-linked genes in region Xq28 since they escape inactivation and might have a role in this disorder. A full clinical and cytogenetic characterization of all POF cases is important to highlight a pattern and help to understand which genes are crucial for normal ovarian development.

## Background

Premature ovarian failure (POF) is an early ovarian dysfunction characterized by the cessation of menses before the age of 40 years [1,2] that affects 1% of women [3]. The diagnosis is established by the presence of FSH (follicle stimulating hormone) serum level higher than 40 mIU/ml [4], detected on at least two occasions a few weeks apart [5]. Although the exact etiology is still unknown, several causes have been associated with POF and may include autoimmunity, infections, iatrogenesis and a strong genetic component, that can vary from single gene alterations to chromosome abnormalities [6].

X;autosome balanced translocations and X-chromosome deletions have been reported in POF patients, leading to the identification of two main critical regions for normal ovarian function on the long arm of this chromosome, specifically at Xq13-q21 [7] and Xq26-q27 [4,8]. In the case of X;autosome balanced translocations, these can either lead to gene disruption at the rearrangement breakpoints, or to a position effect alteration, changing the normal expression of genes involved in ovarian function [9]. X-linked genes known to escape inactivation can also be responsible for the occurrence of POF associated with total or partial monosomies of the X-chromosome, reflecting a situation of haploinsufficiency for those genes [9].

One of the genes known to be associated with POF is *FMR1 *(Fragile X mental retardation), located at Xq27.3 and responsible for Fragile X Syndrome (FXS). It is a form of X-linked mental retardation caused by the expansion of an instable CGG repeat in the 5' untranslated region of the gene [1,10]. The syndrome occurs when the number of the repeats exceeds 200, being denominated as full mutation alleles. This is responsible for hypermethylation and gene inactivation, leading to absence of FMRP (Fragile X mental retardation protein) and, consequently, causing mental retardation [11]. Men with full mutation alleles are always affected, whereas only one third of women are so, due to X-chromosome inactivation [10]. Several studies have been associating *FMR1 *premutation alleles, which may have 55 to 200 CGG repeats, and POF, with approximately 20% of premutation carrier women being affected [11]. Since full mutation carriers do not have an increased risk for ovarian dysfunction, the molecular mechanism underlying the association between POF and premutation alleles, although still unravelled, should not be related to the absence or reduction of FMRP [12].

The present case was referred as part of a study group of women with POF for the evaluation of their karyotypes and association to the *FMR1 *gene CGG repeat number. We report a case of a 29 year old woman with a *de novo *Xq26 to Xqter deletion that includes *FMR1 *gene associated with POF. Besides having only one functional allele prone to suffer inactivation, she has no FXS symptoms.

## Results

### FMR1 repeats determination

**Figure 1:**
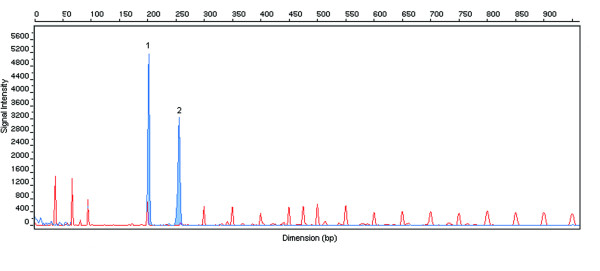
**Electropherogram of *FMR1 *gene CGG repeat number analysis in the patient with Xqter deletion**. The first peak with higher signal intensity (1) corresponds to the X-chromosome gender specific fragment and the second one (2) to the normal *FMR1 *allele with 20 CGG repeats. There is a missing third peak due to the deletion. The remaining peaks with lower intensity correspond to ROX1000 size standard.

*FMR1 *gene CGG repeat number evaluation revealed the presence of only one allele (Figure 1). A woman with a normal karyotype would have three peaks for this analysis, the first one corresponding to the X-chromosome and the other two corresponding to the two alleles of *FMR1 *gene. After repeating the analysis, in order to exclude an amplification failure during PCR reaction, the result was confirmed, being present only one allele with 20 CGG repeats.

### Cytogenetic analysis

**Figure 2:**
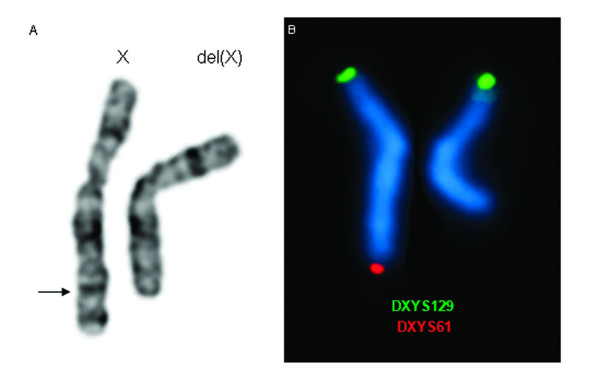
**Conventional and molecular cytogenetics results**. A - Partial karyogram of GTG banded X-chromosomes of the patient. The arrow indicates the region of the breakpoint in the X-chromosome. B - Dual colour FISH with the subtelomeric specific probes showing the normal X-chromosome and the deleted X-chromosome (on the right).

GTG high resolution banded metaphase spreads from the subject were analyzed and revealed a large terminal deletion in the long arm of one of the X-chromosomes in all 10 metaphases studied (Figure 2A). Conventional cytogenetics results suggest a probable deletion breakpoint between bands Xq25-q26, being her final karyotype 46,X,del(X)(q25~q26). The subject's mother karyotype was normal. As the father had already deceased it was not possible to perform cytogenetics analysis.

### Fluorescence in situ hybridization

**Figure 3:**
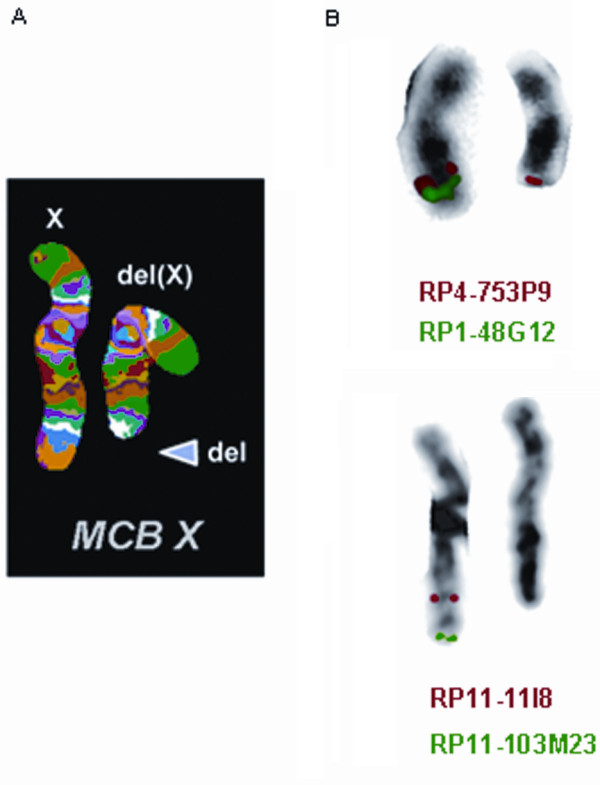
**FISH results**. A - MCB image showing the normal and the deleted X-chromosome. B - Image with the results of the specific hybridization of the BAC clones that delimited the deletion breakpoint (RP4-753P9 is present and RP11-11I8 is absent). Each chromosome pair has is own legend relatively to the clones used and their fluorescence colours.

FISH analysis with the subtelomeric specific probe DXYS61 showed only one signal for Xqter in all metaphases scored, confirming the conventional banding cytogenetic findings (Figure 2B). The integration of MCB and BAC probes results allowed us to conclude with more precision that the deletion breakpoint is at Xq26 (Figure 3). The breakpoint was between 128.660 Mb and 133.964 Mb.

### FMR1 methylation analysis

**Figure 4:**
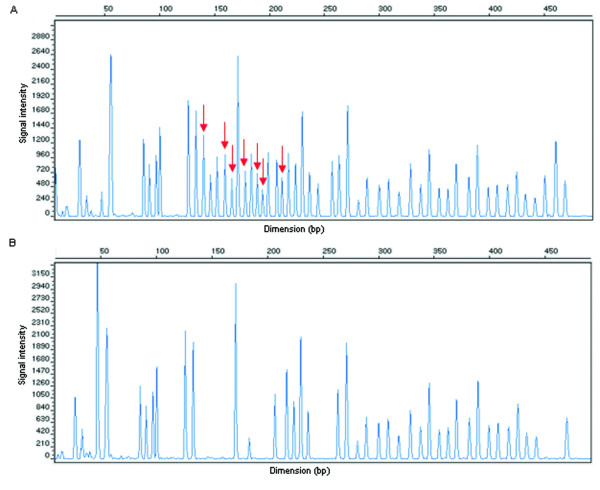
**Electropherograms of MS-MLPA results**. A - Corresponds to the undigested sample, with arrows pointing out probes with *Hha*I restriction site. B - Illustrates the restriction pattern of the digested samples, where probes with *Hha*I restriction site are absent.

This analysis revealed that the X-chromosome subjected to methylation was the one with the qter deletion, as all probes with *Hha*I recognition site were digested, meaning that they were not methylated in the normal allele present (Figure 4). Although visual analysis was quite conclusive, the methylation status was further analyzed with the Coffalyser software which revealed a methylation status of 0% (data not shown). FMR1 gene methylation analysis excluded allele drop out as a possible explanation for the presence of only one allele in the FMR1 CGG repeat number PCR analysis [13]. If two alleles were present, in a homozygous pattern (a) or a normal allele and a full mutation allele (b) the MS-MLPA result would be clearly different. The existence of a second allele would always be detected by the presence of methylation, at a lower percentage due to normal X inactivation (a), or at a higher percentage in due to both X inactivation and full mutation allele methylation (b).

## Discussion

X-chromosome deletions have been associated with POF for more than a decade, with two X-chromosome regions, named POF1 and POF2, mainly associated with POF. POF1 region limits are not consensual among literature, as some authors define it as Xq23-q27 [8], whereas others define it from Xq26 to Xq28 [14]. POF2 region is well established between Xq13.3-q21 [7]. Most of X-chromosome abnormalities associated with POF described in this region are X;autosome balanced translocations, with80% of the chromosome breakpoints disrupting Xq21 [15]. However, women with deletions involving this gene-poor region are not affected by POF, being the most plausible explanation a position effect on autosomal ovary-specific genes translocated to the X-chromosome, and not an involvement of X-linked genes [15].

**Figure 5:**
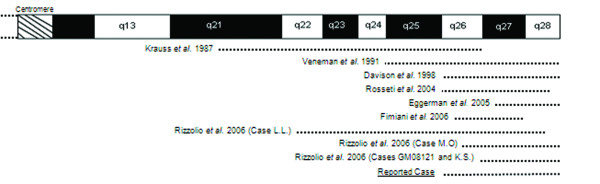
**Schematic representation of Xq**. The dotted lined represent the extension of the deletions described in the literature and of the reported case.

POF1 region deletions are by far more common as being associated with POF, with several cases already described, and, in most of them, the same deletion is present in two or even three generations of the same family. As illustrated in Figure 5, Krauss *et al*. in 1987 reported an interstitial deletion 46,XX,del(X)(pter → q21.3::q27 → qter) in a three generation family affected by POF and in 1991 Veneman *et al*. reported a mother and her daughter, both with POF, and an Xq25 to Xqter deletion [16,17]. Davison *et al*. (1998) published a 46,X,del(X)(q26) karyotype in both mother and daughter with POF at 28 and 26 years old, respectively; Rosseti *et al*. (2004) reported an interstitial deletion 46,X,del(X)(q26 → q28) in two affected women, with secondary amenorrhea at 17 and 22 years old, and her mother, with POF at 43 years old [18,19]. Fimiani *et al*. (2006) also reported the case of both mother and daughter with POF at 43 and 26 years old, respectively, both with a 46,X,del(X)(q26.2q28) karyotype, after the report by Eggerman *et al*. (2005) of a mother and daughter with a 46,X,del(X)(q27.2 or q27.3) karyotype and POF at 36 and 28 years old, respectively [20,21]. The same figure illustrates four additional cases reported by Rizzolio [15]. These reports raise the question that factors other than the deletion might be involved in POF, since women with the same deletion manifest POF at a different age, and some are able to reproduce whereas others do not [19].

In the present case, the mother has a normal karyotype and as the father had already deceased, it was not possible to evaluate if the deletion was *de novo *or inherited from the father. However, this deletion in a male would be incompatible with life, and so, we can safely hypothesize that the deletion is *de novo*.

The absence of Fragile X syndrome features in this woman was quite intriguing to us since, with only one functional allele, which according to Lyon hypotheses should be inactivated in 50% of cells, there would be a reduction to half in FMRP levels [22]. A methylation assay performed to access the methylation status of the unique *FMR1 *allele present resulted in a negative methylation pattern. This allows us to conclude that the normal X-chromosome is active, whereas the deleted X-chromosome was preferentially inactivated, explaining the absence of Fragile X symptoms, as there are normal levels of FMRP. Cells with the abnormal X-chromosome active would have a deficiency in gene products from the deleted region, explaining the negative selection against such cells, resulting in skewed X-chromosome inactivation (XCI) whenever there is a structural abnormality of one of the X homologous [22].

Carrel and Willard (2005) obtained, from fibroblast cells, an expression profile for the genes located in the inactive X-chromosome (X_i_) revealing that about 25% of genes escape inactivation being expressed at different levels and from different regions of the chromosome [23]. One of such clusters of genes expressed from X_i _maps to the gene-rich region Xq28, where the expression level may reach 50% [23].

Altogether, these results suggest that haploinsuffiency of the genes located in the deleted region is a promising explanation for the POF scenario, especially when it involves Xq28. The lack of expression of those deleted genes that normally escape X inactivation may compromise ovarian function. To further evaluate this situation it would be important to perform X inactivation status assays in every women with X-chromosome deletion and POF, and, as if expected, a skewed XCI pattern is observed, this would further support the hypothesis of haploinsufficiency of the deleted genes. MS-MLPA for *FMR1 *gene can be a valuable tool for this assessment, whenever deletion involves Xq27 region, as it is a fast and easy to perform technique, allowing the achievement of precise results. Oral contraceptives prescribed to our patient might have delayed the diagnosis of the POF condition that could have manifested earlier taking into account her menstrual patterns. This suggests that contraceptive therapy provided her the adequate hormonal levels and mitigate the menopausal symptoms due to an eventual ovarian insufficiency, what may imply that genes involved in the deletion can have a function at this level. One of the patients reported by Rosseti *et al*. (2004) has a similar clinical history, as she had irregular menses, started to take pill at 19, and when she stopped, at 22 years old, she had amenorrhea [19]. Yeshaya and colleagues interestingly reported that microdeletion syndromes could be associated with altered replication patterns of genes not associated with the aberrant chromosome [12]. We can not discard that the Xq terminal deletion observed in our patient causes altered replication timing of genes associated with POF. However, if this would be the case, other X-chromosome deletions would be associated with POF. It would be interesting to study the replication time of other patients with POF due to X deletions [24].

The time during development when skewed X-chromosome inactivation takes place can also influence the onset of POF, since woman with the same deletion manifest POF at different ages and sometimes with different severities. Understanding how this happens and which gene(s) are necessary in a double dosage expression can lead to an improvement in reproductive knowledge and to the implementation of strategies to delay or prevent premature ovarian failure, allowing women to preserve her reproductive life.

Nevertheless, it is thought that 5-10% of these women will be able to conceive and will need appropriate advice due to high risk of fragile X syndrome or POF in their descendents [25]. Preconceptual counseling should be offered to those women giving them full disclosure about the risks of transmitting the disease and possible preventive measures [25]. In addition, prenatal testing through amniocentesis or chorionic villus sampling should be recommended for pregnant women carriers of the fragile X mutation or premutation.

Recently new assisted reproduction techniques associated with preimplantation genetic diagnosis (PGD) performed after an in-vitro fertilization cycle (IVF), gave the opportunity for a selection of embryos free of the premutation or full mutation [25].

In our case, at the present time, the only reproductive alternatives that could be undertaken include IVF with oocyte donation or allotransplantation of ovarian tissue.

To summarize, these data reflect how important it is to report all cases with POF and X-chromosome deletions and their complete clinical history, in order to highlight a pattern and try to understand which regions are in fact crucial for normal ovarian development. Special attention should be paid to Xq28 region due to the knowledge that genes on this region escape from methylation in the X_i _and also due to the increased number of POF patients with deletions involving this region.

## Materials and methods

### Subject

A 29 year old woman was referred from the gynecology department of our local maternity due to premature ovarian failure. She had menarche at 11 years, experienced oligomenorrhea and at 14 years old she fulfilled the diagnosis criteria for polycystic ovarian syndrome and received a prescription of oral contraceptives. Trying to get pregnant, she suspended medication 15 years later, but she neither got pregnant or menstruated again. Hormonal analysis revealed an FSH level of 155.5 mIU/ml, LH of 62.7 mIU/ml and 16 pg/ml for E_2_. The patient had no history of autoimmune diseases or surgeries. Her mother did not report a past history of subfertility and experienced menopause at 50 years. This woman is part of a group of patients with premature ovarian failure recruited for a study to access the association between this condition and *FMR1 *gene CGG repeat number. The study was approved by the medical board of the hospital and all participants gave their written informed consent.

### FMR1 gene CGG repeats determination

Genomic DNA was extracted from peripheral blood lymphocytes using Jetquick blood and cell culture DNA Midi Spin kit (Genomed, Löhne, Germany). DNA concentration and purity were measured using a NanoDrop1000 Spectrophotometer (Thermo Scientific, Waltham, USA). The sample was analysed with Abbot Fragile X kit (Abbot, Illinois, USA), which consists of a PCR reaction specific to determine the number of CGG repeats present at *FMR1 *gene. A PCR reaction of 20 μl of final volume containing 13 μl of High GC PCR Buffer, 0.8 μl of *FMR1 *primers, 0.6 μl of gender primers, 1.2 μl of TR PCR enzyme mix and 3 μl of genomic DNA (67 ng/ul) was performed in an Applied Biosystems ABI 2720 Thermal Cycler (Applied Biosystems, Foster City CA, USA). The PCR conditions used were 15 cycles of 98.5°C for 10 s, 58°C for 1 min and 75°C for 6 min, followed by 15 cycles starting at 98.5°C for 10 s, 56°C for 1 min and 75°C for 6 min. PCR products were purified with CleanUp Enzyme mix and the alleles were sized using an automated sequencer ABI Prism 310Genetic Analyser (Applied Biosystems) by comparison with the size standard Rox1000, both products from Abbott (Abbott).

### Cytogenetic analysis

Peripheral blood samples were collected and metaphase chromosomes were prepared according to standard cytogenetic procedures [26]. GTG high resolution banded chromosomes were analyzed using a Nikon Eclipse microscope (Nikon Instruments, Badhoevedorp, Netherlands) coupled with the Cytovision system (Applied Imaging International Lda, Newcastle upon Tyne, UK).

### Fluorescence in situ hybridization

Fluorescence *in situ *hybridization (FISH) was performed according to standard procedures, using specific probes for subtelomeric Xq (DXYS61) and Xp (DXYS129) (Cytocell, Cambridge, UK) chromosomal regions. A total of 10 metaphases were analyzed with a Nikon Eclipse fluorescence microscope (Nikon) coupled with the Cytovision system (Applied Imaging International Lda).

To further characterize the breakpoint, multicolour banding (MCB) was performed using seven partial chromosome painting (pcp) probes described in Weise *et al*. 2008 [27]. The 10 metaphases scored were analysed using a Zeiss Axioplan fluorescence microscope (Zeiss, Jena, Germany) with MetaSystems (Isis) software (Altlussheim, German). Six BAC (bacterial artificial chromosomes) clones were also used in order to clarify the breakpoint. The analysis software was the same used for MCB. Table 1 summarizes the BAC clones used and their location on the X-chromosome.

**Table 1:** Characteristics of the BAC clones used for FISH analysis

Clone	Start (Mb)	End (Mb)	Location	Insert Size (bp)
RP4-753P9	128,544	128,660	Xq25	116560

RP11-11I8	133,964	134,153	Xq26.3	188357

RP1-48G12	141,595	141,794	Xq27.3	199015

RP11-103M23	153,345	153,519	Xq28	174233

Built at the UCSC Genome Browser, version NCBI3537/hg17 and accessed at June 26^th ^2010.

### FMR1 methylation analysis

Methylation-Specific Multiplex Ligation-Dependent Probe Amplification (MS-MLPA) was performed using the SALSA MS-MLPA MEO29-B1 kit (MRC-Holland, Amsterdam, Netherlands). Genomic DNA, 300 ng, was mixed with 1 μl of denaturation buffer and MLPA protocol was performed according to the manufacturers' instructions. PCR reactions were carried out on an Applied Biosystems ABI 2720 Thermal Cycler (Applied Biosystems). Samples were analysed by capillary electrophoresis on an ABI Prism 310 Genetic Analyser (Applied Biosystems) and Genescan software (Applied Biosystems) was used to extract the quantitative data. These were analyzed with Coffalyser analysis software (MRC-Holland, Amsterdam, Netherlands) to determine methylation status.

The aim of this analysis was to determine the degree of methylation of the only *FMR1 *allele present, localized on the normal X-chromosome. MS-MLPA uses the methylation-sensitive restriction enzyme *Hha*I to determine the degree of methylation, by comparing digested and undigested samples of the same patient. Unmethylated probes with *Hha*I recognition site will be digested, and hence will disappear in the sample subject to digestion, whereas methylated probes won't be digested and, thus, will be present after PCR amplification. As the subject has only one allele, the degree of methylation of *FMR1 *gene is indicative of the degree of methylation of the normal X-chromosome.

## Competing interests

The authors declare that they have no competing interests.

## Authors' contributions

SIF- Participated in the design of the study, carried out the molecular biology work and drafted the manuscript.

EM- Participated in the design of the study and carried out the subtelomeric FISH experiments.

MP - Carried out the cytogenetic work.

JA - Contributed with clinical information.

TL - Carried out MCB and BAC-FISH experiments and revised critically the manuscript.

JBM- Has been involved in the design of the study and drafting of the manuscript, revising it critically for important intellectual content.

IMC - Coordinated and conceived the study, being involved in the critical revision of the manuscript for important intellectual content.

All authors have read and approved the final manuscript.
